# Sex differences in the association between diabetes and hypertension and the risk of stroke: cohort of the Tehran Lipid and Glucose Study

**DOI:** 10.1186/s13293-022-00421-7

**Published:** 2022-03-15

**Authors:** Azra Ramezankhani, Donna Parizadeh, Fereidoun Azizi, Farzad Hadaegh

**Affiliations:** 1 Prevention of Metabolic Disorders Research Center, Research Institute for Endocrine Sciences, Shahid Beheshti University of Medical Sciences, Tehran, Iran; 2 Endocrine Research Center, Research Institute for Endocrine Sciences, Shahid Beheshti University of Medical Sciences, Tehran, Iran; 3 Prevention of Metabolic Disorders Research Center, Research Institute for Endocrine Sciences (RIES), Shahid Beheshti University of Medical Science, Number 24, Yemen Street, Shahid Chamran Highway, P.O. Box: 19395-4763, Tehran, Iran

**Keywords:** Diabetes, Hypertension, Sex, Stroke, Ischemic

## Abstract

**Background:**

We assessed the sex differences in the association between blood pressure categories and glucose intolerance status with overall and ischemic stroke among Iranian adults.

**Methods:**

This prospective study was conducted on 5349 individuals (2446 men) aged ≥ 40 years. Cox models were used to estimate adjusted hazard ratios (HRs) and men-to-women ratios of HRs (RHRs) of overall and ischemic stroke for systolic (SBP) and diastolic (DBP) blood pressure, pre-hypertension, hypertension, fasting plasma glucose (FPG), pre-diabetes and type 2 diabetes (T2D).

**Results:**

Each 0.55 mmol/L increase in FPG was associated with a higher HR of overall stroke in men than women [RHRs 1.05 (1.01–1.09)]. The associations between each 10 mmHg increase in DBP and stroke events were stronger in men than women, with RHRs of 1.20 (1.00–1.45) and 1.29 (1.04–1.60) for overall and ischemic stroke, respectively. Hypertension was associated with a higher HR of overall [RHRs: 2.41 (1.21–4.8)] and ischemic stroke [2.37 (1.12–5.01)] in men than women. We also found that T2D *was associated* with *higher risks* of overall and ischemic stroke in men than women: the RHRs were 2.16 (1.24–3.75) and 1.93 (1.05–3.55) for overall and ischemic stroke, respectively.

**Conclusion:**

Hypertension and T2D induced higher risk of overall and ischemic stroke in men than women among Iranian population.

**Supplementary Information:**

The online version contains supplementary material available at 10.1186/s13293-022-00421-7.

## Highlights

The associations between diastolic blood pressure and stroke events were stronger in men than women.

The fasting blood plasma level was associated with higher risk of stroke events in men than women.

Hypertension and diabetes were associated with higher risk of stroke events in men than women.

The association between systolic blood pressure with ischemic stroke was higher in men than women.

**Supplementary Information:**

The online version contains supplementary material available at 10.1186/s13293-022-00421-7.

## Background

Stroke is the second leading cause of death worldwide, responsible for the 5.5 million deaths attributed to this cause in 2016 [1]. Although the incidence of stroke is declining in high-income countries, 70% of strokes occur in low- and middle-income countries, warranting the need to gain knowledge about the risk factors in these regions [2–4]. Hypertension and type 2 diabetes (T2D) are known as major risk factors of stroke [4]. It has been shown that hypertension alone contributes to about 50% of the burden of stroke [5] and patients with T2D have more than twofold increased risk of stroke [6].

Sex-difference has been observed in the incidence rate and risk factors of stroke, with discrepancies in results among different populations. According to the Global Burden of Diseases (GBD) report from 1990 to 2016, the age-specific stroke incidence was significantly higher among men above 55 years than their female counterparts, but was similar in both sexes before 55 years of age [7]. Recently, the American Heart Association’s 2021 Heart Disease and Stroke Statistics Update reported that age-specific incidence rates of stroke are substantially lower in women than men in younger- and middle-aged groups [8].

A meta-analysis study of 64 cohorts suggested that women with T2D were at substantially higher risk of coronary heart disease (CHD) than men with T2D [9]. However, several prospective cohort studies found no meaningful differences between men and women in the association between T2D and stroke [10, 11]. Recently, a study has shown that T2D and hypertension were associated with a 25% and 36%, respectively, higher risk of stroke in women than in men [3]. As most studies are based on high-income country data, uncertainty remains regarding significant sex difference in stroke risk factors in low- and middle-income countries.

Given the observed increase in the stroke incidence in low- and middle-income countries during the last four decades [2], and the sex difference in prevalence of some modifiable CVD risk factors [12–14] in these countries, the aim of this study was therefore to assess the sex-difference in the association between T2D and hypertension, as major stroke risk factors [15], and the risk of incident stroke among participants of the Tehran Lipid and Glucose Study (TLGS).

## Methods

### Study population

The TLGS is an ongoing prospective and population-based cohort study designed to investigate the risk factors and outcomes for non-communicable diseases (NCDs). The first phase of TLGS initiated in 1999 on 15,005 people aged ≥ 3 years in a representative population of Tehran, capital of Iran. The second phase, started in 2002 in which 3550 new participants were also added. Re-examinations were conducted in intervals of 3 years for all participants which has been continued to date [16]. Detailed explanations about study population, baseline measurement and follow-up visits have been fully described elsewhere [17]. In the present study, we included 6308 participants aged ≥ 40 years from the first (*n* = 5303) and second (*n* = 1005) phases and followed them annually until the end of the study (20 March 2018). Of these, we excluded 100 individuals who had a history of stroke events, 568 people who did not have follow-up data, and 291 people who had missing value on covariates at baseline. Therefore, a total of 5349 (2446 men) participants (86% of eligible) were enrolled in this work (Additional file 1: Fig. S1).

### Measurements

Baseline demographic data including age, sex and education level, and also smoking status, medication use, history of cardiovascular disease (CVD) or family history of premature CVD (FH-CVD) were collected through a standardized questionnaire. The body weight and height were measured using the standard protocols, and body mass index (BMI) was calculated as weight (kg)/height (m^2^). Systolic and diastolic blood pressure (SBP, DBP) were measured with the mercury column sphygmomanometer and average of two consecutive measurements on the same arm, after at least 5 min of seated rest in a chair, was calculated [17]. Fasting blood samples were taken from all participants in the morning to measure the total cholesterol (TC), fasting plasma glucose (FPG) and 2-h post-challenge plasma glucose (2 h-PCPG) using the enzymatic colorimetric method [17].

### Definition of terms

The FH-CVD was positive if any female first degree relative < 65 years, or any male first degree relative < 55 years had history of CVD. Blood pressure (BP) was categorized as the normal (SBP < 120 mmHg and DBP < 80 mmHg), pre-hypertension (SBP 120–140 mmHg or DBP 80–90 mmHg) and hypertension (SBP ≥ 140 mmHg or DBP ≥ 90 mmHg or taking antihypertensive drugs) [18]. We defined three categories for T2D status: no-diabetes (FPG < 5.55 mmol/L and 2-hPCPG < 7.77 mmol/L), pre-diabetes (5.55 ≤ FPG < 7 mmol/L or 7.77 ≤ 2 h-PCPG < 11.1 mmol/L) and T2D (FPG ≥ 7 mmol/L or 2-hPCPG ≥ 11.1 mmol/L or the use of anti-diabetes drugs). Current smoking was defined as having smoked cigarettes or other smoking implements (water-pipes, pipes) daily or occasionally. Education was defined in three levels: < 6 years, 6–12 years and ≥ 12 years of education.

### Outcome

The primary outcomes were the incidence of all stroke and fatal or nonfatal ischemic stroke. Details on the collection of outcomes in TLGS have been published previously [19]. In brief, all participants were followed annually via telephone calls, starting from inclusion in the study and ended on March 20, 2018, date of death, or the first stroke. Outcome confirmation for incident stroke was conducted by an adjudication committee consisting of a principal investigator, an internist and an epidemiologist [19, 20]. Incident stroke was defined as all cases of definite and possible stroke and transient ischemic attack (TIA). Definite stroke was defined according to the WHO definition as “rapidly developing clinical signs of or global disturbance of cerebral function, lasting > 24 h or leading to death with no apparent cause other than that of vascular origin” [21], or imaging of acute clinically relevant brain injuries accompanied by quickly vanishing symptoms. Possible stroke was defined as an episode of acute focal neurological deficit without imaging indicative of stroke or when data did not fully qualify for the definition of definite stroke. Transient ischemic accident (TIA) was considered when clinical symptoms resolved in < 24 h. Cases that were not primarily hemorrhagic based on medical record and imaging results were defined as ischemic stroke.

### Statistical methods

We used descriptive statistics to compare baseline characteristics between men and women and also, between participants and non-participants. Non-participants included people with missing data at baseline or without any follow-up data. For each outcome the crude incidence rates and 95% confidence interval (CI) per 1000 person-years was calculated. Moreover, in a Cox proportional hazard model, we estimated the men-to-women risk of stroke adjusted for age, BMI, hypertension, T2D, smoking status, TC, educational level, history of CHD, FH-CVD and lipid lowering drugs.

Cox regression models were also used to examine the association of BP categories and glucose intolerance status with the end points adjusted for age (age adjusted) and also a set of confounders (multivariable adjusted). Multivariable model for diabetes was adjusted for BMI, TC, smoking status, hypertension, education, FH-CVD, history of CHD, and lipid lowering drugs. For FPG in continuous form, we further adjusted for anti-diabetes drugs and the results are shown for each 0.55 mmol/L (10 mg/dL) increase. In multivariable models for hypertension, we adjusted for BMI, TC, smoking status, T2D, education, FH-CVD, history of CHD, and lipid lowering drugs. For SBP and DBP, models were further adjusted for antihypertensive drugs and the hazard ratios (HRs) of endpoints are shown for each 20 and 10 unit increase in SBP and DBP, respectively.

In all regression models we included an interaction term of each risk factor (T2D, hypertension and their components) with sex to estimate the men-to-women ratios of HRs (RHRs) for that risk factor.

In a sensitivity analysis, hypertensive patients were allocated to two groups according to treatment with antihypertensive drugs, and the association between BP status (normal, prehypertension, untreated and treated hypertensives) and outcomes were examined.

In other sensitivity analysis, all models were repeated by excluding individuals with history of CVD at baseline. All statistical analyses were performed using R version 3.6.2 and 2-sided *P* values < 0.05 were considered as statistically significant.

## Results

The baseline characteristics of the study population (*n* = 5349; 46% men) at baseline are reported in Table 1. The mean (SD) age at baseline were 55.2 (10.5) and 52.9 (9.2) years in men and women, respectively. Overall, the levels of SBP, DBP, FPG, BMI, and TC, and the prevalence of T2D, hypertension, and FH-CVD were significantly lower in men than women. However, compared to women, men were more likely to smoke, have higher level of education and history of CHD, but less likely to use antihypertensive, anti-diabetes and lipid lowering medications.

**Table 1:** Baseline characteristics of the study population, Tehran Lipid and Glucose Study (1999–2018)

	Men (*n* = 2446)	Women (*n* = 2903)	*P* value
Age, year	55.2 (10.5)	52.9 (9.2)	< 0.001
SBP, mmHg	125.9 (20.8)	127.6 (21.3)	0.005
DBP, mmHg	79.4 (11.9)	80.9 (11.1)	< 0.001
FPG, mmol/L	5.8 (1.9)	5.9 (2.4)	< 0.001
BMI, kg/m^2^	26.2 (3.9)	29.2 (4.6)	< 0.001
TC, mmol/L	5.4 (1.1)	6.0 (1.2)	< 0.001
Blood pressure status (%)			
No hypertension	834 (34.1)	812 (28.0)	< 0.001
Pre-hypertension	836 (34.2)	963 (33.2)	
Hypertension	776 (31.7)	1128 (38.9)	
Glucose tolerance status (%)			
No diabetes	1596 (65.2)	1839 (63.3)	0.013
Pre-diabetes	528 (21.6)	599 (20.6)	
Type 2 diabetes	322 (13.2)	465 (16.0)	
Smoking status (%)			< 0.001
Past and never	1744 (71.3)	2778 (95.7)	< 0.001
Current	702 (28.7)	125 (4.3)	
Education level (%)			
< 6 years	1040 (42.5)	1927 (66.4)	< 0.001
6–12 years	1051 (43.0)	857 (29.5)	
> 12 years	355 (14.5)	119 (4.1)	
Drug use (%)			
Antihypertensive drugs	216 (8.8)	515 (17.7)	< 0.001
Lipid lowering drugs	80 (3.3)	220 (7.6)	< 0.001
Anti-diabetes drugs	146 (6.0)	263 (9.1)	< 0.001
Family history of CVD (%)	339 (13.9)	558 (19.2)	< 0.001
History of CHD (%)	210 (8.6)	175 (6.0)	< 0.001

Data are shown as mean ± standard deviation (SD) or number (percent) and numbers (percentage) as appropriate

*SBP* systolic blood pressure; *DBP* diastolic blood pressure; *BMI* body mass index; *FPG* fasting plasma glucose; *TC* total cholesterol; *CVD* cardiovascular diseases; *CHD* coronary heart disease

From the population with T2D (*n* = 787), 409 subjects (51.9%) had been on glucose lowering drugs at first examination. From these subjects, 317 (77.5%), 12 (2.9%) and 26 (6.4%) persons had used sulfonylureas, metformin and insulin, respectively. The remaining 54 participants had missing information on glucose lowering drugs type. Moreover, 44 (10.7%) participants had used two types of glucose lowering drugs. Among the 787 diabetic subjects, women were more likely than men to use anti-diabetes medications (56.6% vs. 45.3%, *P* = 0.002), however, there was no significant difference in the type of anti-diabetes drugs between men and women (*P* = 0.588).

Also, among the hypertensive patients (*n* = 1904), 731 (38.3%) individuals had used antihypertensive drugs. Of this individuals, 399 (54%) had used only one type of antihypertensive drugs including beta-blockers (39.2%), angiotensin converting enzyme inhibitors (ACEIs)/angiotensin-receptor blockers (ARBs) (4.6%), vasodilators (4.1%), calcium channel blockers (CCBs) (3.8%) and diuretic agents (2.7%). Moreover, of 731 participants, 25.7, 8.1, and 1.9% subjects had used two, three and four types of antihypertensive drugs, respectively. The remaining 9.7% participants had used other types of antihypertensive drugs or had no information on drugs type. Among the 1904 hypertensive individuals, the use of antihypertensive drugs was higher in women than men (45.7% vs. 27.8%, *P* < 0.001). Moreover, taking beta-blockers were more common in women than in men (71.5% vs. 61.2%, *P* < 0.05). But, taking ACEIs/ARBs and CCBs were higher in men than in women (35.0% vs. 21.2% and 32.2% vs. 20.7%, respectively, *P* < 0.01).

Additional file 1: Table S1 displays a comparison between the baseline characteristics of participants and non-participants. Participants were younger and had lower levels of SBP and FPG compared to non-participants. They were also less likely to smoke, have T2D and history of CHD, and use lipid lowering drugs than non-participants.

Among all participants, and during a median follow-up of 17.9 years (interquartile range: 13.5–18.4) years, 292 (128 women) incident cases of stroke were documented, including 237 (101 women) cases of ischemic stroke. Among total study population, the crude incidence rates for overall stroke and ischemic stroke were 3.6 (3.2–4.0) and 2.9 (2.5–3.3) per 1000 person-years, respectively. The corresponding values were 2.8 (2.4–3.4) and 2.2 (1.8–2.7) for women and 4.5 (3.9–5.3) and 3.7 (3.1–4.4) for men. After adjusting for multiple confounders, the risk of overall stroke and ischemic stroke were also higher in men than in women (relative risk ratio 1.76; 95% CI 1.33–2.33 and 1.78; 1.31–2.42, respectively).

### Blood pressure status

The age-adjusted HRs of all studied risk factors and the men-to-women RHRs for overall and ischemic stroke are presented in Additional file 1: Tables S2 and S3.

**Fig. 1:**
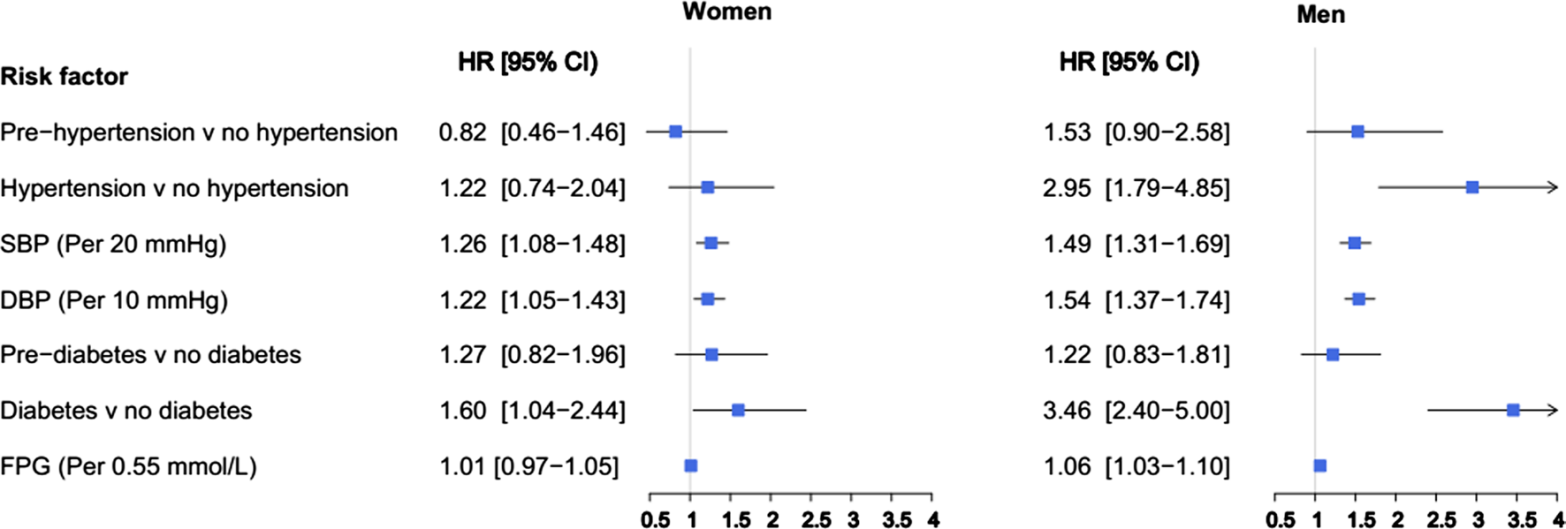
Multivariable adjusted HRs for incident stroke associated with risk factors. *SBP* systolic blood pressure; *DBP* diastolic blood pressure; *FPG* fasting plasma glucose; *HR* hazard ratio; *CI* confidence interval

**Fig. 2:**
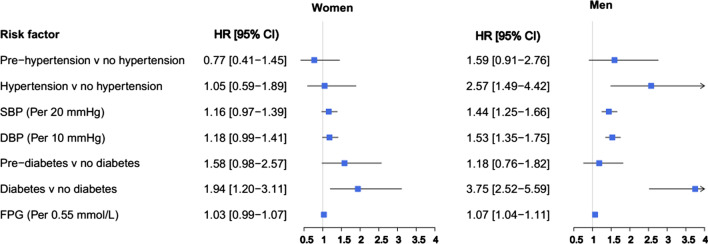
Multivariable adjusted HRs for incident ischemic stroke associated with risk factors. *SBP* systolic blood pressure; *DBP* diastolic blood pressure; *FPG* fasting plasma glucose; *HR* hazard ratio; *CI* confidence interval

Figures 1 and 2 present the results of multivariable models. Accordingly, increased levels of SBP and DBP were associated with an increased risk of overall stroke in both sexes; the multivariable adjusted HRs associated with each 20 mmHg increase in SBP were 1.26 (1.08–1.48) and 1.49 (1.31–1.69) in women and men, respectively. The corresponding values for each 10 mmHg increase in DBP were 1.22 (1.05–1.43) and 1.54 (1.37–1.74) (Fig. 1).

The increased levels of SBP and DBP were associated with a higher risk of ischemic stroke in men only, with HRs of 1.44 (1.25–1.66) and 1.53 (1.35–1.75) for SBP and DBP, respectively (Fig. 2). However, among women, a marginally significant association was found for DBP with the risk of ischemic stroke (HRs of 1.18 (0.99–1.41, *P* = 0.058).

**Fig. 3:**
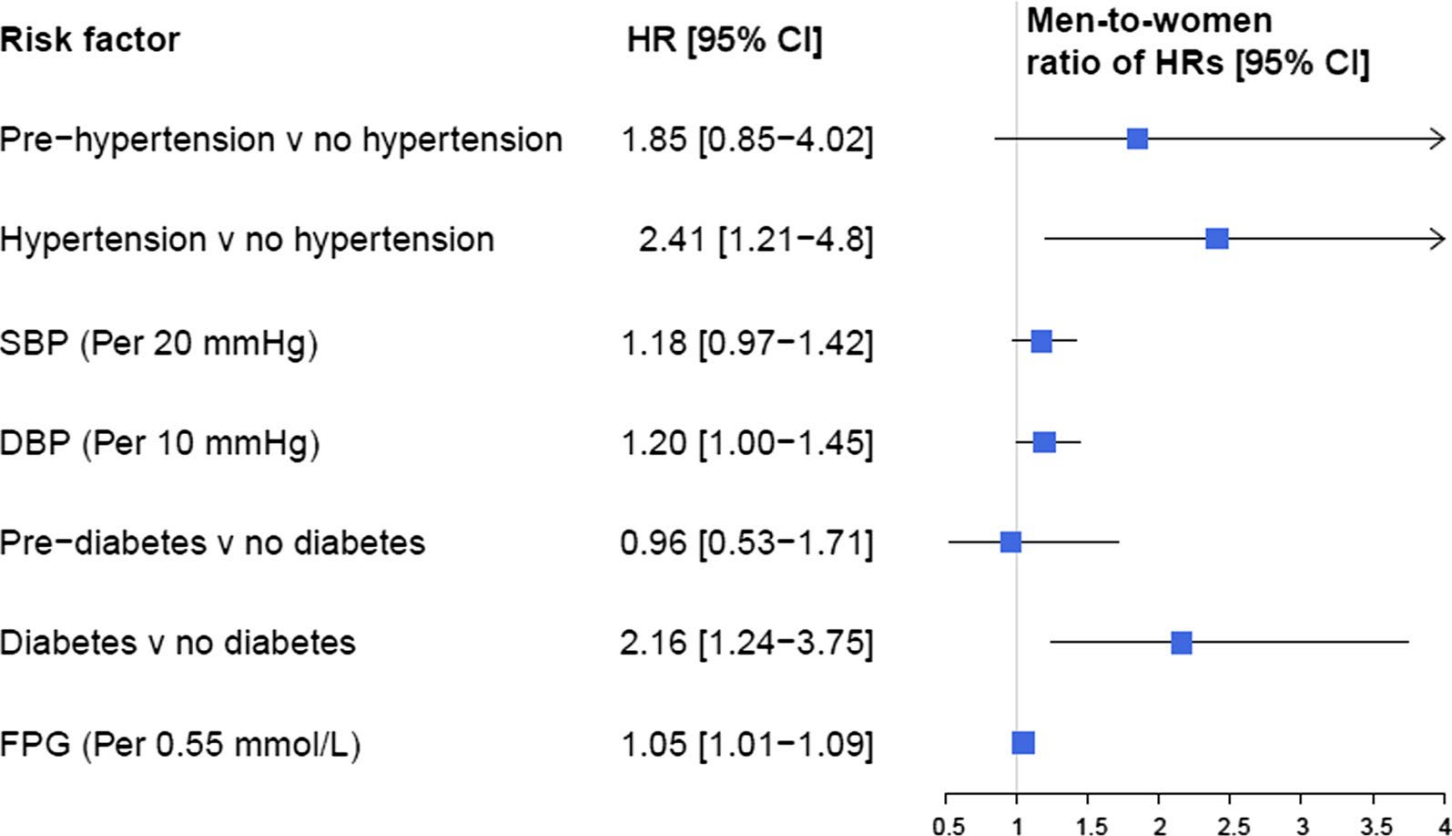
Multivariable adjusted men-to-women ratios of HRs for association between risk factors and incident stroke. *SBP* systolic blood pressure; *DBP* diastolic blood pressure; *FPG* fasting plasma glucose; *HR* hazard ratio; *CI* confidence interval

**Fig. 4:**
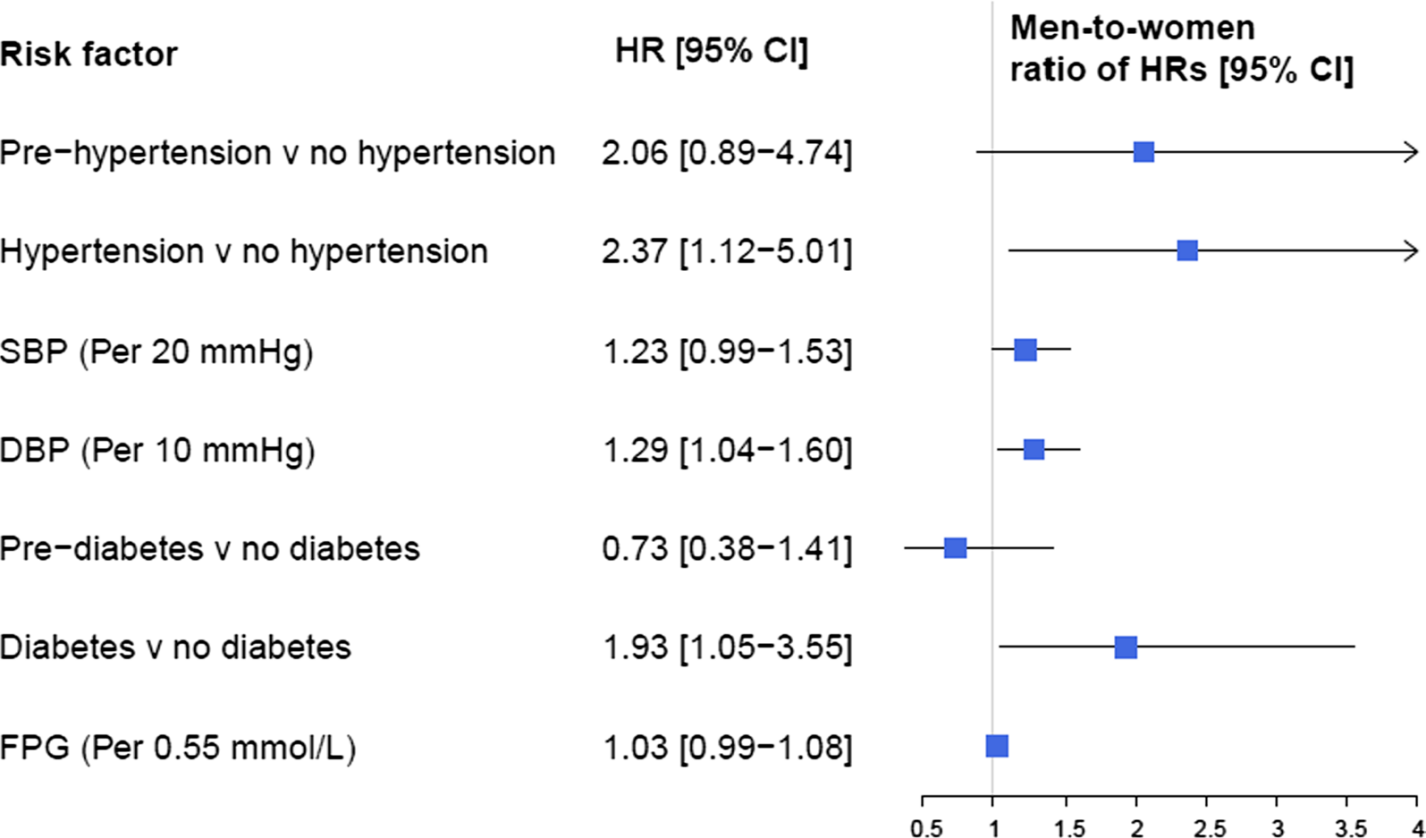
Multivariable adjusted men-to-women ratios of HRs for association between risk factors and incident ischemic stroke. *SBP* systolic blood pressure; *DBP* diastolic blood pressure; *FPG* fasting plasma glucose; *HR* hazard ratio; *CI* confidence interval

We found a significant sex difference in the associations between DBP with overall and ischemic stroke (men-to-women RHRs of 1.20 (1.00–1.45) and 1.29 (1.04–1.60) for overall and ischemic stroke, respectively) (Fig. 3). Moreover, sex difference tended to be significant for the association between increased SBP with risk of only ischemic stroke (men-to-women RHRs of 1.23 (0.99–1.53), *P* = 0.056) (Fig. 4).

Hypertension was associated with a substantially higher risk of overall and ischemic stroke only in men: the multivariable adjusted HRs of overall and ischemic stroke associated with hypertension were 2.95 (1.79–4.85) and 2.57 (1.49–4.42), respectively; and the men-to-women RHRs were 2.41 (1.21–4.8) and 2.37 (1.12–5.01) for overall and ischemic stroke, respectively (Figs. 3 and 4).

### Glucose tolerance status

In multivariable models, each 0.55 mmol/L (10 mg/dL) increase in FPG was associated with increased risk of overall and ischemic stroke in men only, with HRs of 1.06 (1.03–1.10) for overall stroke and 1.07 (1.04–1.11) for ischemic subtype (Figs. 1 and 2). We found evidence of a sex difference in the associations between increased FPG with overall stroke in the multivariable adjusted models with a men-to-women RHRs of 1.05 (1.01–1.09) (Fig. 3).

We also found marginally significant association between pre-diabetes and risk of ischemic stroke only among women (HR 1.58 (0.98–2.57), *P* = 0.059).

Results from multivariable models showed that T2D was associated with the substantially higher risks of overall and ischemic stroke, in both women and men. The HRs were 1.60 (1.04–2.44) in women and 3.46 (2.40–5.00) in men for overall stroke (Fig. 1), with a men-to-women RHRs of 2.16 (1.24–3.75) (Fig. 3). The corresponding values were 1.94 (1.20–3.11) in women and 3.75 (2.52–5.59) in men (Fig. 2) for ischemic stroke, with the men-to-women RHRs of 1.93 (1.05–3.55) (Fig. 4).

### Sensitivity analysis

The results of the sensitivity analysis are presented in Additional file 1: Tables S4 and S5, in which we categorized hypertensives into two groups according their antihypertensive treatment. In the confounder-adjusted model, both treated and untreated hypertensive men had higher risk for overall and ischemic stroke, compared with normal BP men, with the nearly the same HRS. There was evidence of the sex difference in the associations between treated and untreated hypertension with overall and ischemic stroke with a higher HR for men.

When individuals with history of CVD at baseline were excluded, the pattern of sex-specific HRs and also the men-to-women RHRs approximately remained the same as those in the main analysis (Additional file 1: Tables S6 and S7).

## Discussion

In this population-based study among 5349 middle-aged men and women from the Middle East, hypertension was associated with more than two times higher risk for both overall and ischemic stroke in men than women.

T2D *was associated* with *approximately twofold* and over two*fold higher risks* of ischemic and overall stroke, respectively, in men than women.

Each 10 mmHg increase in DBP was associated with an almost 20% higher risk of both overall and ischemic stroke in men, and each 0.55 mmol/L increase in FPG was associated with 5% greater risk of overall stroke in men than women.

We found the incidence of stroke to be higher among men than women, in accordance with the global reports [22]; however, considerably greater in both sexes compared with most western countries. The excessive incidence of stroke has been noted before in other studies from Iran [23]. The underlying reason is not clear yet, but the high prevalence, unawareness, as well as poor compliance in the management of important risk factors such as T2D, hypertension [24], and dyslipidemia [23], ethnic disparities, and socioeconomic status may be important contributing factors [25].

Our findings build upon a recently expanding literature on the sex-specific associations of major cardiometabolic risk factors with incident stroke. Such sex-differences may be explained by underlying biological mechanisms or environmental and behavioral factors [26].

A systematic review of 124 cohorts in 2013 demonstrated similar impact of SBP increments (each 10-mmHg SBP rise) on cardiovascular outcomes in both sexes [27]. This was supported later by two large cohort studies of 471,971 and 1.25 million participants from the UK which reported a lack of sex-difference in the risk of stroke induced by rising SBP or DBP [28, 29]. Inconsistent with previous studies, we found that raised DBP was associated with a considerably greater risk of stroke events in men than women.

Although hypertension is recognized as a major risk factor for stroke among both sexes [15], there are discrepancies in the evidence regarding sex-differences in this associations. Among the UK biobank [28] and the INTERSTROKE study participants [5], hypertension was associated with a higher risk of stroke in women than men. In contrast, our analysis showed that the risk of stroke events was substantially greater in hypertensive men (either untreated or treated) than hypertensive women. Interestingly, we found the similar risk for stroke events among treated and untreated hypertensive individuals vs. non-hypertensives ones that might be related to low prevalence of controlled hypertension, poor compliance and inadequate dosage of antihypertensive drugs among Iranian population [24].

Several epidemiological studies have examined the effect of FPG on stroke events, but the findings are inconclusive [30]. Some studies have shown the sex difference in the association between FPG levels and stroke. For example, a study conducted by the DECODE Study Group found a strong positive association between FPG and stroke mortality in women but not in men [31]. However, most other studies did not separate men and women. In our study, we found that the excess risk of stroke associated with raised FPG was greater in men than women, a finding that was in line with our previous study, in which results of sex-stratified analysis showed that FPG of 6.1–6.9 mmol/L was associated with increased risk of stroke only among men [32].

T2D is a major risk factor for stroke. A meta-analyses including 64 cohorts found that excess risk of stroke associated with T2D was 27% greater in women than men after adjusting for conventional risk factors [9]. Subsequent studies have reported mixed findings on this association; for example, a large-scale electronic health record-based study in UK including about 2 million patients found no sex-difference in the association between T2D and stroke subtypes [33]. However, recently, an analysis of UK Biobank data have demonstrated that women with T2D had an almost 25% higher relative risk of stroke compared with men [28]. In the present study, we found the higher risk of stroke events associated with T2D among men than women.

The lower risk of stroke induced by hypertension and T2D among women than men in this study can be largely attributed to the sex disparities in the sociobehavioral and environmental factors. Sex disparities in health care and disease control among different populations have been previously suggested as a main contributor in the outcomes of hypertension and T2D [9, 26–28]. On the other hand, several previous studies have demonstrated sex-differences in the rate of cardiovascular risk factor control among patients with T2D or hypertension. Accordingly, the rate of BP control among hypertensive patients has been reported higher among women in Europe [34, 35], but similar to men in the United States [36]. However, women with T2D were less likely to achieve cardiovascular risk factors goals than men in studies from the United States [37] and Europe [38]. In our study, women were more likely than men to use anti-hypertensive and anti-diabetes medications. Moreover, we have previously demonstrated that women with and without T2D had a positive trend in the rate of BP control and a decreasing mean of DBP from 1999 to 2011; whereas, BP control was not improved significantly among men and DBP mean decreased only among those men without T2D during the same time [39]. In addition, a national study in Iran found that the estimated national control of hyperglycemia and hypertension was better among women than men [40]. Therefore, better cardiovascular risk factor control may contribute to the lower risk of stroke among women with T2D and hypertension than their male counterparts in this study.

Moreover, occupational stress and job strain has been demonstrated to increase the risk of stroke [41]. A review of the findings from Europe, USA, and Japan indicated 10–40% excess risk of CHD and stroke with exposure to psychosocial work stress [42]. According to the national census in Iran, women’s share of work force has been as low as 11% in 2011, with about 2 million employed women and 14 million housewives in the country at the time [43]. This substantial sex difference in employment rate and the consequent sex-difference in the level of experienced occupational stress may have exposed men to a higher risk of cardiovascular outcomes in the past decade in Iran.

In addition, both short-term and long-term exposure to air pollution has been recognized as an important modifiable risk factor for CVD including stroke [44]. We have previously highlighted a positive association between high levels of air pollution and CVD in the TLGS population, which was significant among the under 60 years [45]. Considering the abovementioned employment rates and social structure of Iran, men are likely to spend more time outdoors, exposing them to high levels of air pollutants.

Other factors that may explain the lower risk of stroke among women with T2D and hypertension includes alcohol use. According to the INTERSTROKE study, even low levels of alcohol use can increase the risk of stroke as much as 14% in both men and women [5]. Recently, a systematic review from Iran indicated that men were almost 2.5 times more likely than women to drink alcohol; a sex difference that is considerably larger than the corresponding global estimates and probably related to the socio-cultural patterns in our community [46]. Therefore, although we could not adjust our results for alcohol use due to lack of data, the higher relative risk of stroke among men with T2D and hypertension may be attributed to the higher alcohol consumption among Iranian men than women.

In addition to the abovementioned factors, there is evidence of sex-difference in the intrinsic pathways regulating BP and the cardiovascular system [26, 47]. These mechanisms tend to shift towards cardioprotection and delay the progression of CVD in women; however, the protection attenuates after menopause [47]. Of note, about 60% of the women in our study population were menopausal or post-menopausal, making it reasonable to assume that the sociobehavioral and environmental factors are the main contributors to the lower relative risk of stroke among women with T2D and hypertension than men.

## Strengths and limitations

The main strength of this study is that it is based on a longitudinal population-based study, with a large sample and long-term follow-up. An extensive range of baseline characteristics of the participants were measured by laboratory studies or physical examinations by trained staff and the diagnosis of stroke was confirmed by an adjudication committee. Nevertheless, some limitations to our study need to be acknowledged. First, the number of events for stroke subtypes was too limited to estimate sex-specific associations. Second, our data on smoking and medication use were self-reported, which introduces measurement bias that could be differential by sex. Third, due to the observational nature of this study, residual confounders remain despite adjustment for a wide range of priori-selected confounders. Finally, considering the potential role of psychosocial and environmental factors, the results of this study may not be generalizable to populations from other regions; however, considering the differences between our results with those from other countries [27, 28], we believe that it is important to consider sex-differences in the association of hypertension and T2D with stroke in the context of cultural and structural differences of each region.

## Perspectives and significance

Our findings provide new information about sex-difference in the associations of BP and glucose intolerance with stroke among a middle-aged population from the Middle East. Men were at a considerably higher risk of stroke induced by hypertension and T2D, compared with women. Psychosocial and environmental factors may contribute to the observed sex-difference and need to be further studied. Treatment and prevention strategies tailored according to sex could improve outcomes for T2D and hypertension.

## Supplementary Information

**Additional file 1: Figure S1.** Flowchart of sample selection for the study. **Table S1.** Baseline characteristics of respondents and non-respondents, Tehran Lipid and Glucose Study (1999–2018). **Table S2.** Age adjusted HRs for incident stroke associated with risk factors, by sex. **Table S3.** Age adjusted HRs for incident ischemic stroke associated with risk factors. **Table S4.** Multivariable adjusted HRs for incident stroke associated with blood pressure categories. **Table S5.** Multivariable adjusted HRs for incident ischemic stroke associated with blood pressure categories. **Table S6.** Multivariable adjusted HRs for incident stroke associated with risk factors. **Table S7.** Multivariable adjusted HRs for incident ischemic stroke associated with risk factors.

## Data Availability

The datasets analyzed during the current study are available from the corresponding author on reasonable request.
