# Supplementary material for: Sex differences in the association between diabetes and hypertension and the risk of stroke: cohort of the Tehran Lipid and Glucose Study
Source: Biol Sex Differ. 2022 Mar 15;13:10. doi: 10.1186/s13293-022-00421-7 (PMC8922930; doi:10.1186/s13293-022-00421-7)
Supplement: Supplementary file 1 — Additional file 1: Figure S1. Flowchart of sample selection for the study. Table S1. Baseline characteristics of respondents and non-respondents, Tehran Lipid and Glucose Study (1999–2018). Table S2. Age adjusted HRs for incident stroke associated with risk factors, by sex. Table S3. Age adjusted HRs for incident ischemic stroke associated with risk factors. Table S4. Multivariable adjusted HRs for incident stroke associated with blood pressure categories. Table S5. Multivariable adjusted HRs for incident ischemic stroke associated with blood pressure categories. Table S6. Multivariable adjusted HRs for incident stroke associated with risk factors. Table S7. Multivariable adjusted HRs for incident ischemic stroke associated with risk factors. [file 13293_2022_421_MOESM1_ESM.docx]

**ADDITIONAL MATERIAL**

6308 participants aged ≥ 40 years,

5303 recruited from Phase 1 (1999-2002)

, and 1005 recruited from Phase 2 (2002-2005)

Exclusion:

History of stroke at baseline, n=100

6208 participants eligible at baseline

Exclusion:

People with no follow-up data, n=568

People with follow-up data, n=5640

Exclusion:

Missing values in covariates, n=291

Final study sample, n=5349 (2446 men)

**Additional Figure S1:** Flowchart of sample selection for the study

| **Additional Table S1.** Baseline characteristics of respondents and non-respondents, Tehran Lipid and Glucose Study (1999-2018) | | | |
| --- | --- | --- | --- |
|  | **Non-respondents**  **n=859** | **Respondents**  **n=5349** | **P value** |
| Age, year | 55.3 (10.9) | 53.9 (9.9) | 0.001 |
| SBP, mmHg | 128.6 (22.2) | 126.8 (21.1) | 0.039 |
| DBP, mmHg | 80.5 (11.2) | 80.2 (11.4) | 0.411 |
| FPG, mmol/L | 6.1 (2.6) | 5.9 (2.2) | 0.020 |
| BMI, kg/m^2^ | 27.9 (4.6) | 27.8 (4.5) | 0.720 |
| TC, mmol/L | 5.7 (1.2) | 5.7 (1.2) | 0.300 |
| Blood pressure status (%) |  |  |  |
| No hypertension | 200 (27.4) | 1646 (30.8) | 0.146 |
| Pre-hypertension | 250 (34.2) | 1799 (33.6) |  |
| Hypertension | 280 (38.4) | 1904 (35.6) |  |
| Glucose tolerance status (%) |  |  |  |
| No diabetes | 437 (61.3) | 3435 (64.2) | 0.009 |
| Pre-diabetes | 140 (19.6) | 1127 (21.1) |  |
| Diabetes | 136 (19.1) | 787 (14.7) |  |
| Smoking status (%) |  |  |  |
| Past and never | 595 (80.5) | 4522 (84.5) | 0.006 |
| Current | 144 (19.5) | 827 (15.5) |  |
| Education level (%) |  |  |  |
| <6 years | 492 (58.1) | 2967 (55.5) | 0.315 |
| 6-12 years | 280 (33.1) | 1908 (35.7) |  |
| >12 years | 75 (8.9) | 474 (8.9) |  |
| Drug use (%) |  |  |  |
| Antihypertensive drugs | 130 (15.1) | 731 (13.7) | 0.243 |
| Lipid lowering drugs | 66 (7.7) | 300 (5.6) | 0.019 |
| Anti-diabetic drugs | 76 (8.8) | 409 (7.6) | 0.218 |
| Family history of CVD (%) | 146 (17.0) | 897 (16.8) | 0.883 |
| History of CHD (%) | 87 (10.1) | 385 (7.2) | 0.004 |
| Data are shown as mean ± standard deviation (SD) or number (percent) as appropriate.  **SBP**: systolic blood pressure; **DBP**: diastolic blood pressure; **BMI**: body mass index; **FPG**: fasting plasma glucose; **TC**: total cholesterol; **CVD**: cardiovascular diseases; **CHD**: coronary heart disease | | | |

| **Additional Table S2:** Age adjusted HRs for incident stroke associated with risk factors, by sex | | | |
| --- | --- | --- | --- |
|  | **Men**  **(n=2446)** | **Women**  **(n=2903)** | **Men-to-women ratios of HRs** |
| **Hypertension** |  |  |  |
| Pre-hypertension *v* no hypertension | 1.59 (0.95-2.67) | 0.94 (0.53-1.66) | 1.69 (0.78-3.65) |
| Hypertension *v* no hypertension | 3.21 (1.99-5.19) | 1.61 (0.98-2.66) | 1.99 (1.00-3.93) |
| **SBP** (Per 20 mmHg) | 1.54 (1.36-1.74) | 1.38 (1.19-1.60) | 1.11 (0.92-1.34) |
| **DBP** (Per 10 mmHg) | 1.57 (1.40-1.76) | 1.30 (1.13-1.51) | 1.20 (1.00-1.45) |
|  |  |  |  |
| **Glucose tolerance status** |  |  |  |
| Pre-diabetes *v* no diabetes | 1.39 (0.95-2.05) | 1.45 (0.94-2.23) | 0.96 (0.53-1.71) |
| Diabetes *v* no diabetes | 3.89 (2.71-5.57) | 1.98 (1.31-3.00) | 1.96 (1.13-3.40) |
| **FPG** (Per 0.55 mmol/L) | 1.09 (1.06-1.12) | 1.05 (1.02-1.08) | 1.04 (1.00-1.08) |
| **SBP**: systolic blood pressure; **DBP**: diastolic blood pressure; **FPG**: fasting plasma glucose; **HR**: hazard ratio | | | |

| **Additional Table S3:** Age adjusted HRs for incident ischemic stroke associated with risk factors | | | |
| --- | --- | --- | --- |
|  | **Men**  **(n=2446)** | **Women**  **(n=2903)** | **Men-to-women**  **ratios of HRs** |
| **Blood pressure status** |  |  |  |
| Pre-hypertension *v* no hypertension | 1.62 (0.94-2.79) | 0.88 (0.47-1.65) | 1.83 (0.80-4.19) |
| Hypertension *v* no hypertension | 2.80 (1.68-4.66) | 1.50 (0.86-2.59) | 1.86 (0.89-3.90) |
| **SBP** (Per 20 mmHg) | 1.48 (1.29-1.70) | 1.30 (1.10-1.54) | 1.13 (0.92-1.40) |
| **DBP** (Per 10 mmHg) | 1.55 (1.36-1.76) | 1.26 (1.07-1.49) | 1.23 (0.99-1.51) |
|  |  |  |  |
| **Glucose tolerance status** |  |  |  |
| Pre-diabetes *v* no diabetes | 1.31 (0.85-2.03) | 1.78 (1.10-2.87) | 0.74 (0.38-1.41) |
| Diabetes *v* no diabetes | 4.16 (2.82-6.13) | 2.41 (1.51-3.84) | 1.72 (0.94-3.16) |
| **FPG** (Per 0.55 mmol/L) | 1.10 (1.07-1.12) | 1.07 (1.03-1.10) | 1.03 (0.98-1.07) |
| **SBP**: systolic blood pressure; **DBP**: diastolic blood pressure; **FPG**: fasting plasma glucose; **HR**: hazard ratio | | | |

| **Additional Table S4:** Multivariable adjusted HRs for incident stroke associated with blood pressure categories | | | |
| --- | --- | --- | --- |
| **Blood pressure categories** | **Men**  **(n=2446)** | **Women**  **(n=2903)** | **Men-to-women ratios of HRs** |
| Pre-hypertension *v* no hypertension | 1.53 (0.90-2.59) | 0.82 (0.46-1.46) | 1.85 (0.85-4.02) |
| Untreated hypertension *v* no hypertension | 2.96 (1.77-4.94) | 1.15 (0.65-2.02) | 2.57 (1.22-5.43) |
| Treated hypertension *v* no hypertension | 2.97 (1.65-5.34) | 1.30 (0.75-2.25) | 2.28 (1.05-4.95) |

| **Additional Table S5:** Multivariable adjusted HRs for incident ischemic stroke associated with blood pressure categories | | | |
| --- | --- | --- | --- |
| **Blood pressure categories** | **Men**  **(n=2446)** | **Women**  **(n=2903)** | **Men-to-women ratios of HRs** |
| Pre-hypertension *v* no hypertension | 1.59 (0.91-2.76) | 0.77 (0.41-1.45) | 2.06 (0.89-4.74) |
| Untreated hypertension *v* no hypertension | 2.59 (1.49-4.50) | 1.04 (0.55-1.94) | 2.49 (1.10-5.64) |
| Treated hypertension *v* no hypertension | 2.82 (1.49-5.32) | 1.19 (0.65-2.18) | 2.37 (1.02-5.51) |

| **Additional Table S6.** Multivariable adjusted HRs for incident stroke associated with risk factors | | | |
| --- | --- | --- | --- |
|  | **Men***  **n=2236** | **Women***  **n=2728** | **Men-to-women ratios of HRs** |
| **Blood pressure status** |  |  |  |
| Pre-hypertension *v* no hypertension | 1.48 (0.84-2.60) | 0.87 (0.48-1.58) | 1.70 (0.75-3.84) |
| Hypertension *v* no hypertension | 3.32 (1.94-5.67) | 1.24 (0.72-2.12) | 2.68 (1.28-5.59) |
| **SBP (Per 20 mmHg)** | 1.54 (1.35-1.76) | 1.27 (1.08-1.51) | 1.20 (0.98-1.47) |
| **DBP (Per 10 mmHg)** | 1.57 (1.38-1.78) | 1.26 (1.08-1.48) | 1.24 (1.02-1.51) |
|  |  |  |  |
| **Glucose tolerance status** |  |  |  |
| Pre-diabetes *v* no diabetes | 1.26 (0.83-1.90) | 1.26 (0.80-1.98) | 1.00 (0.54-1.83) |
| Diabetes *v* no diabetes | 3.24 (2.19-4.81) | 1.45 (0.92-2.30) | 2.23 (1.23-4.04) |
| **FPG (Per 0.55 mmol/L)** | 1.06 (1.03-1.09) | 1.00 (0.96-1.05) | 1.05 (1.00-1.10) |
| *Study population included participants without history of CVD at baseline.  **SBP**: systolic blood pressure; **DBP**: diastolic blood pressure; **FPG**: fasting plasma glucose; **HR**: hazard ratio; **CVD**: cardiovascular disease; **FH-CVD**: family history of CVD. | | | |

| **Additional Table S7.** Multivariable adjusted HRs for incident ischemic stroke associated with risk factors | | | |
| --- | --- | --- | --- |
|  | **Men***  **n=2236** | **Women***  **n=2728** | **Men-to-women**  **ratios of HRs** |
| **Blood pressure status** |  |  |  |
| Pre-hypertension *v* no hypertension | 1.58 (0.87-2.87) | 0.80 (0.42-1.52) | 1.97 (0.82-4.70) |
| Hypertension *v* no hypertension | 2.92 (1.64-5.21) | 1.10 (0.61-1.97) | 2.65 (1.20-5.86) |
| **SBP** (Per 20 mmHg) | 1.48 (1.27-1.72) | 1.17 (0.97-1.41) | 1.26 (1.00-1.59) |
| **DBP** (Per 10 mmHg) | 1.55 (1.35-1.79) | 1.20 (1.00-1.44) | 1.29 (1.03-1.61) |
|  |  |  |  |
| **Glucose tolerance status** |  |  |  |
| Pre-diabetes v no diabetes | 1.18 (0.74-1.89) | 1.66 (1.01-2.70) | 0.71 (0.36-1.39) |
| Diabetes v no diabetes | 3.52 (2.29-5.41) | 1.75 (1.05-2.91) | 2.01 (1.04-3.86) |
| **FPG** (Per 0.55 mmol/L) | 1.07 (1.04-1.11) | 1.03 (0.98-1.07) | 1.04 (1.00-1.09) |
| *Study population included participants without history of CVD at baseline.    **SBP**: systolic blood pressure; **DBP**: diastolic blood pressure; **FPG**: fasting plasma glucose; **HR**: hazard ratio; **CVD**: cardiovascular disease; **FH-CVD**: family history of CVD. | | | |
